# Neuroinflammation in comorbid depression in Alzheimer's disease: A pilot study using post-mortem brain tissue

**DOI:** 10.1016/j.nsa.2024.104051

**Published:** 2024

**Authors:** Jordan T. Lin, Mizuki Morisaki, Srisharnitha A. Sampathkumar, Laurie C. Lau, Delphine Boche, Golam M. Khandaker, Lindsey I. Sinclair

**Affiliations:** a Dementia Research Group, University of Bristol, Bristol, UK; b Clinical and Experimental Sciences, Faculty of Medicine, Sir Henry Wellcome Laboratories, University of Southampton, Southampton, UK; c Clinical and Experimental Sciences, Faculty of Medicine, University of Southampton, Southampton, UK; d MRC Integrative Epidemiology Unit, Population Health Sciences, Bristol Medical School, University of Bristol, Bristol, UK; e NIHR Bristol Biomedical Research Centre, Bristol, UK; f Avon and Wiltshire Mental Health Partnership NHS Trust, Bristol, UK

**Keywords:** Alzheimer's disease, Depressive disorder, Inflammation, Cytokines, Tumour necrosis factor alpha, Interleukin 4

## Abstract

Comorbid depression and Alzheimer's disease (AD) is associated with poorer prognosis than either condition alone. Neuroinflammation has been implicated in the pathogenesis and progression of both depression and AD, but much of the existing research has been based on peripheral blood immune markers. Relatively little is known about the neuroinflammatory environment when these conditions occur simultaneously and using immune measures directly in the brain tissue. This pilot study aimed to examine brain inflammatory marker changes in AD cases comparing those with and without comorbid depression.

Post-mortem brain tissue from AD cases with depression (n = 23) and AD cases with no history of psychiatric illness (n = 25) were analyzed for a range of inflammatory markers, including markers of microglial function (Iba1, P2RY12, CD64 and CD68 measured by immunohistochemistry); endothelial inflammatory markers (ICAM-1 and VCAM-1 measured by ELISA); and cytokine levels (IFN-γ, IL-1β, IL-2, IL-4, IL-6, IL-8, IL-10, IL-12p70, IL-13, and TNF-α measured using Mesoscale Discovery Multiplex Assays).

Brains of AD cases with comorbid depression, compared with AD alone, had increased IL-4 in the superior frontal gyrus and increased TNFα & IL-12p70 in the insula. Levels of all other inflammatory markers including markers of microglial function and endothelial inflammation were similar between the two groups.

We found no consistent changes in cytokines between the two brain regions in individuals with comorbid depression in AD. Further work in larger cohorts is needed to understand brain region specificity of immune marker alterations and the relationship of these changes with pre-mortem clinical outcomes.

## Highlights

• It is unclear why some individuals with AD develop depression. Current treatments are ineffective for depression in AD.
• We investigated whether neuroinflammation is involved in depression in AD using brain tissue from people with AD +/− depression.
• We measured 10 different cytokines and two downstream markers of the effect of neuroinflammation on blood vessels in the brain.
• We found no consistent differences between the two brain areas that we studied, suggesting neuroinflammation is not the cause of depression in AD.

## Abbreviations

Aβ: Amyloid beta
AD: Alzheimer's disease
APOE: Apolipoprotein E
APP: Amyloid precursor protein
BDNF: Brain-derived neurotrophic factor
BSA: Bovine serum albumin
CD64: Cluster of differentiation 64
CD68: Cluster of differentiation 68
CRP: C-Reactive protein
CSDD: Cornell scale for depression in dementia
EDTA: Ethylenediaminetetraacetic acid
ELISA: Enzyme-linked immunosorbent assay
GDS: Geriatric depression scale
Iba1: Ionized calcium binding adaptor molecule 1
ICAM-1: Intercellular adhesion molecule 1
IFN-γ: Interferon-gamma
IHC: Immunohistochemistry
IL: Interleukin
MMSE: Mini-mental state examination
MSD: Meso Scale Discovery
NFT: Neurofibrillary tangles
P2RY12: Purinergic receptor P2Y12
PBS: Phosphate-buffered saline
RPM: Revolutions per minute
TNF-α: Tumour necrosis factor alpha
VCAM-1: Vascular cell adhesion molecule 1

## 1 Introduction

Alzheimer's disease (AD) is a neurodegenerative disorder characterised by progressive loss of memory, language, visuospatial and cognitive ability (World Health Organization, 2021; Reitz and Mayeux, 2014). Patients frequently suffer from non-cognitive symptoms such as depression, aggressiveness and irritability (Hussain et al., 2020; Vik-Mo et al., 2018). Depression (MDD) is clinically defined as a persistent feeling of sadness and/or hopelessness as well as a loss of interest or pleasure in activities, leading to a change in functioning for two weeks or greater (World Health Organization, 2021). The prevalence of depression is increased in people living with AD and an Increased prevalence of depression in AD populations and increased risk of dementia in those with a history of depression have been reported with some authors suggesting that later life depression may be an early sign of AD (Asmer et al., 2018; Khundakar and Thomas, 2015; Zhao et al., 2016; Ownby et al., 2006; Ma, 2020; Roberto et al., 2021). The comorbidity of AD and depression has been associated with a faster rate of cognitive decline, increased mortality, and a higher rate of institutionalisation (Cerejeira et al., 2012; Ritchie et al., 1998; Burns et al., 1991), re-emphasizing the importance of diagnosing and treat depression, although this is much challenging in the context of AD (Novais and Starkstein, 2015; Orgeta et al., 2017; Banerjee et al., 2011).

Whilst the underlying neurobiological mechanisms of depression and AD are complex, neuroinflammation has been associated in the development and pathogenesis of both conditions (Hussain et al., 2020; Khundakar and Thomas, 2015; Hayley et al., 2021; Troubat et al., 2021; Köhler et al., 2017, 2018). There is strong evidence (reviewed in (Heneka et al., 2015) and (Wong-Guerra et al., 2023)) that neuroinflammation is involved in the pathogenesis of Alzheimer's disease. Observational studies indicate an increased prevalence of depressive disorders in individuals with chronic inflammatory conditions (Patten, 2001; Mikocka-Walus et al., 2016). In a meta-analysis of 82 case-control studies, elevated levels of serum circulating cytokines, among them: interleukin- 1 receptor antagonist (IL-1Ra) and interleukin- 6 (IL-6), were found in individuals diagnosed with depression compared to healthy controls (Köhler et al., 2017). Few previous human *post-mortem* brain tissue studies assessing neuroinflammation in depression have been performed, most of the participants were middle aged or younger and a limited range of cytokines were measured in the protein based studies (Pandey et al., 2012; Tonelli et al., 2008; Mahajan et al., 2018; Clark et al., 2016; Labonté et al., 2017; Shelton et al., 2011). They have revealed alterations in cytokine levels in individuals who died by suicide, including IL-4 (Tonelli et al., 2008), IL-13 (Tonelli et al., 2008), IFNγ (Clark et al., 2016) and TNF-α (Clark et al., 2016). In a meta-analysis IL-6, IL-8 and TNf-α were elevated in the CSF of individuals with depression (Enache et al., 2019).

It is possible that neuroinflammation may underlie depression in AD, but little research has been done. One previous study (n = 275) which used the NPI to rate neuropsychiatric symptoms reported raised serum TNF-α in individuals with depression (Holmes et al., 2011). This was replicated in a smaller study (n = 92) which also identified elevations in serum IL-6 in those with depression in AD compared to AD without depression and controls (Khemka et al., 2014). In AD, neuroinflammation is a recognised key feature, contributing to the progression of the pathology (Chen et al., 2016; Calsolaro and Edison, 2016; Haijun et al., 2019) and cognitive decline (Edison et al., 2008). There is emerging evidence from mendelian randomisation studies that neuroinflammation may play a causal role in AD. (Pagoni et al., 2022) Microglial activation, in AD affected brain regions, as measured by TSPO-PET has been associated with neuropsychiatric symptoms in AD including depression and was a stronger predictor of these symptoms than either amyloid or tau (Aguzzoli C, data presented at AAIC, 2023). We hypothesized that neuroinflammation may be worse in individuals with depression in AD (Hayley et al., 2021; Mukhara et al., 2020).

To explore neuroinflammation in co-morbid depression in Alzheimer's disease, we conducted a pilot human *post-mortem* brain tissue study in which cases were selected on the basis of the presence or absence of depression in the presence of AD. We investigated whether the neuroinflammatory environment was affected in the context of co-morbid depression and AD. As both AD and depression have been studied singly compared to controls and we wished to focus on the unique differences in depression in AD we did not include a healthy control group.

Previous work by our group (Asby et al., 2021) has shown that multiple cytokines were elevated in temporal cortex in AD compared to controls without dementia. Other groups have reported similar findings in different brain areas including in the prefrontal cortex (Tennakoon et al., 2022; Ferguson et al., 2020). We therefore chose in this current pilot study to focus on those with AD with and without depression. We hypothesized that pro-inflammatory cytokines and the activity of microglia, the immune cells of the brain, would be elevated in those with depression and AD, compared to AD alone.

## 2 Methods

### 2.1 Cases

Tissue was sourced from the South West Dementia Brain Bank (Bristol, UK). Groups were age, gender and post-mortem delay matched as far as possible. The brain areas studied were the superior frontal gyrus medial segment and anterior insula. We chose to study these regions because previous neuroimaging studies focusing on depression in AD (Sinclair and Ballard, 2023; Karavasilis et al., 2017) have suggested differential atrophy in these regions in co-morbid depression in AD. This pilot study was part of a larger study looking at gene expression in depression in AD.

#### 2.1.1 Inclusion and exclusion criteria

All cases had a clinical diagnosis of AD prior to death and satisfied *post-mortem* neuropathological consensus criteria for AD (Hyman et al., 2012). The cases were selected on the basis of the presence (n = 23) or absence (n = 25) of depression. Presence of depression either immediately prior to or at any time after onset of AD was defined by a clinical diagnosis of depression *ante-mortem*, geriatric depression scale score ≥8; or Cornell scale for depression in dementia (CSDD) ≥8. Individuals with evidence of other significant brain pathologies or other significant mental health problems (e.g. schizophrenia) were excluded from both groups. In the AD no depression group those with a history of depression and who had been prescribed antidepressant drugs other than for pain were excluded. Individuals were excluded from the depression in AD group if they had had episodes of depression in the past more than 2 years prior to their dementia diagnosis. Information on whether individuals had taken anti-inflammatory, anti-depressant or anti-psychotic medication in the last 12 months of their life were retrieved from the reports as this might modulate neuroinflammation.

#### Ethical approval

This work was carried out under the South West Dementia Brain bank generic ethical approval (ref 18/SW/0029). Relatives of donors provide informed consent at the point of donation for the tissue to be used in peer reviewed research.

### 2.2 Immunohistochemistry

Formalin-fixed paraffin embedded sections were stained for microglial markers associated with a specific function including: Iba1, a widely expressed microglial protein involved in motility (Ohsawa et al., 2004), P2RY12 a purinergic receptor involved in motility and recognised as a key homeostatic marker (Gómez Morillas et al., 2021), CD64 a Fcγ receptor with a high affinity for immunoglobulin (IgG) (Minett et al., 2016), considered as a marker of the systemic immune system involvement, and CD68 a marker of phagocytosis (Minett et al., 2016).

Sections were stained in batches with each batch containing cases from both groups to ensure comparability of immunolabelling. Sections were pre-treated with the appropriate antigen retrieval method antibody which for Iba1, CD68 and P2RY12 involved heated citrate buffer and for CD64 heated EDTA incubation. The primary antibodies incubation was followed by the incubation with the secondary antibodies. Details of primary and secondary antibodies, along with incubation times are presented in Table s1. Staining was revealed by incubation with 3,3′-diaminobenzadine (DAB, Vector Laboratories) chromogen, followed by immersion in copper sulphate solution to enhance antibody staining, before counterstaining with haematoxylin.

### 2.3 Quantification

Quantification of staining was done blinded to the case designation. For each section, 15 random fields were selected in the same anatomical region, and image capture in the pre-defined area acquired at magnification ×20 using the software package Image Pro Plus 7 (Media Cybernetics, MD, USA). The percentage of the image stained with a specific marker was obtained and expressed as the fraction stained in each field (%). Each field was given equal weighting and the mean field fraction was calculated for each section (Minett et al., 2016).

### 2.4 Sample homogenisation

Fresh frozen brain tissue was homogenised in chilled RIPA lysis buffer (Merck) containing protease inhibitor (complete MINI protease inhibitor, Merck) and phosphatase inhibitor (phosSTOP, Merck). Homogenisation was performed using a Precellys homogeniser for (2 × 15 s at 6000 g) with 6–10 zirconia beads in a 2 ml homogenate tube. The homogenates were then centrifuged for 15 min at 13,000 g at 4 °C. Supernatant was aliquoted into non-binding 96-well storage plates (ThermoFisher Scientific) and frozen at −80 °C until required. Total protein was measured for all samples using the Coomassie protein assay kit (Thermo Scientific).

### 2.5 ELISA

To assess blood-brain barrier permeability, ELISAs were carried out on frozen tissue for the human endothelial markers ICAM-1 and VCAM-1 (R&D Systems) using the manufacturer protocols. Samples were diluted 1:200 for ICAM-1 and 1:80 for VCAM-1. Absorbance was measured at 450 nm with the FLUOstar OPTIMA microplate reader (BMG Labtech). Absolute protein levels were then determined using Optima data analysis software to interpolate against the relevant standard curve. Both ICAM-1 and VCAM-1 ELISA were duplicated to ensure reproducibility.

### 2.6 Multiplex assay

Inflammatory proteins were measured on frozen tissue via the V-Plex MesoScale Discovery (MSD) electrochemiluminescence multi-spot assay platform (MesoScale Diagnostics) using the Proinflammatory Panel 1 Human Kit (cat. no. K15049D) to detect: IFN-γ, IL-1β, IL-2, IL-4, IL-6, IL-8, IL-10, IL-12p70, IL-13, and TNF-α. Brain homogenates (1:2 dilution) were used according to the manufacturer's protocol and incubated overnight. A recombinant protein was used for each cytokine as a positive control and to generate a standard curve from which concentrations of each cytokine could be interpolated for each samples. Each plate was imaged on the Meso Quickplex SQ120 (MSD) to obtain absolute target protein level (pg/ml) adjusted for total protein. Samples for which the luminescence was below the lowest standard were classed as below the detection threshold. Using IL-6 as an example the concentration range that could be measured in the tissue homogenates was 0.06–488 pg/mL.

### 2.7 Statistical analysis

Normality was tested with the Shapiro-Wilk's test. ANOVA test for parametric data and Mann Whitney *U* test for non-parametric data were used accordingly. If significant, a posthoc analysis or a pairwise comparison was performed. Any demographic variable which varied between groups (i.e. age, *post-mortem delay, antidepressant use, anti-inflammatory drug use, sex and comorbid conditions*) was included as a covariate. A threshold p value of 0.05 was considered significant. Statistical analysis was carried out using SPSS, JASP Team (2022,. Version 0.16.3) and the graphs were prepared in GraphPad Prism 9.4.

## 3 Results

We were able to obtain tissue from 23 individuals with AD and co-morbid depression and 25 matched cases with AD without depression (see Table 1). The vast majority of individuals in this study had Braak stages of 5/6 and a high CERAD plaque burden. There was no evidence of a between group difference in any of the markers of AD neuropathological burden. Five of the cases with AD without depression were taking antidepressants in the year prior to their demise, as were 17 of the AD with depression group (p < 0.001). The presence of anti-inflammatory medication in the last 12 months of life did not differ between both groups, while those in the AD with depression group were less likely to have had a co-morbid inflammatory illness (p = 0.004). There was no strong evidence of a between group difference in post-mortem interval (p = 0.0683). There was no statistical evidence of a between group difference in *APOE* genotype. For many individuals the last recorded MMSE was a significant time prior to their demise, as individuals became too cognitively impaired to be able to complete the MMSE. There was no MMSE score available for 14 individuals. As shown in Table 1, the vast majority of individuals included in this study had AD of at least moderate severity based on their last MMSE score and there was no evidence of a between group difference. In the AD alone group the 5 individuals taking antidepressants were not also taking antipsychotic medications.

**Table 1:** Demographic data of the cohort used for the investigation of co-morbid depression in Alzheimer's disease.

	AD without depression (AD only) n = 25	AD with depression (AD + MDD) n = 23	Statistical analysis (χ2 test, ANOVA or Mann Whitney *U* test)
Sex: *Male: Female*	10:15	13:10	Χ^2^ = 1.310, p = 0.252
Age at death (years), Mean ± SD	82.8 (7.8)	78.7 (9.9)	p = 0.116
Duration of AD (years)	7.3 (4.2)	8.7 (3.3)	p = 0.285
Mean ± SD			
Last documented MMSE score	14.8 (8.0)	15.1 (6.8)	p = 0.939
Mean ± SD			
Braak stage			Χ^2^ = 2.140, p = 0.544
*III*	2	1	
*IV*	1	1	
*V*	8	12	
*VI*	14	9	
*APOE* genotype			
2/3	2	0	
2/4	3	2	
3/3	7	5	
3/4	5	5	
4/4	0	4	Χ^2^ = 6.510, p = 0.164
Thal phase			
*Unknown*	1	0	
*3*	2	0	
*4*	15	18	
*5*	7	5	Χ^2^ = 3.529, p = 0.317
LATE stage			
*Unknown*	24	22	
*Negative*	1	0	
*2*	0	1	Χ^2^ = 2.007, p = 0.367
Braak lewy body stage			
*Unknown*	7	9	
*Unclassifiable*	1	0	
*0*	17	13	
*2*	0	1	Χ^2^ = 2.705, p = 0.439
CERAD			
*No plaques*	1	0	
*Sparse*	1	1	
*Moderate*	1	4	
*High*	22	18	Χ^2^ = 3.122, p = 0.373
*Post-mortem* delay (hours)			
Mean ± SD	30.2 (14.6)	40.4 (20.0)	p = 0.0683
Use of antidepressant medication in last 12 months			
*No*	20	6	
*Yes*	5	17	Χ^2^ = 14.025, p < 0.001
Use of antipsychotic medication in last 12 months			
*No*	20	16	
*Yes*	5	7	Χ^2^ = 0.696, p = 0.404
Use of anti-inflammatory medication in last 12 m			
*No*	7	10	
*Yes*	16	13	Χ^2^ = 0.840, p = 0.359
Evidence of co-morbid inflammatory condition			
*No*	6	15	
*Yes*	19	8	Χ^2^ = 8.270, p = 0.004

### 3.1 Cytokine expression

**Fig. 1:**
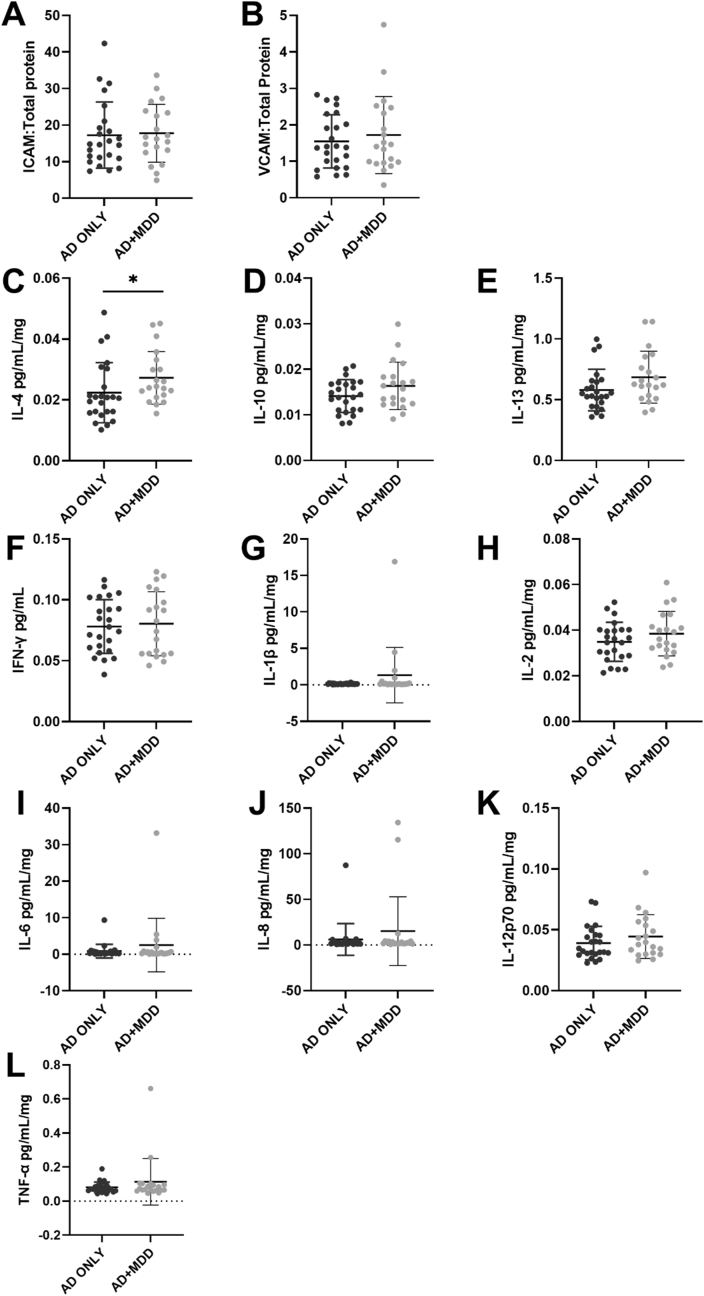
Expression of endothelial cell activation markers and cytokines in superior frontal gyrus of AD and AD + MDD groups. There was no evidence of a between group difference in ICAM-1 (A), VCAM-1 (B) or any of the pro-inflammatory cytokines under investigation (F–L). IL-4 was increased in the AD + MDD group (C) but there was no between group difference in IL-10 (D) or IL-13(E). Please note that all analyses included covariates except for IL-1β, IL-8 and IL-6 which were not normally distributed.

In the superior frontal gyrus medial segment, IL-4 was higher in AD + MDD group (0.028 ± 0.011) compared to AD only (0.022 ± 0.008) (F_1,34_ = 4.186, p = 0.049, Fig. 1C), suggesting that the co-morbidity depression in AD may induce an anti-inflammatory environment. However, none of the other anti-inflammatory cytokines showed significant difference between the two groups (IL-10: F_1,34_ = 1.534, p = 0.224 and IL-13: F_1,34_ = 1.537, p = 0.224, Fig. 1D–E). There was no between group difference in concentrations of the pro-inflammatory cytokines (IFN-γ: F_1, 34_ = 1.421, p = 0.242, IL-1β: W = 265, p = 0.567, IL-2: F_1,34_ = 1.182, p = 0.285, IL-6: W = 275, p = 0.42, IL-8: W = 287, p = 0.276, IL-12p70: F_1,34_ = 0.637, p = 0.43 and TNFα: F_1,34_ = 0.881, p = 0.354; Fig. 1F-L).

**Fig. 2:**
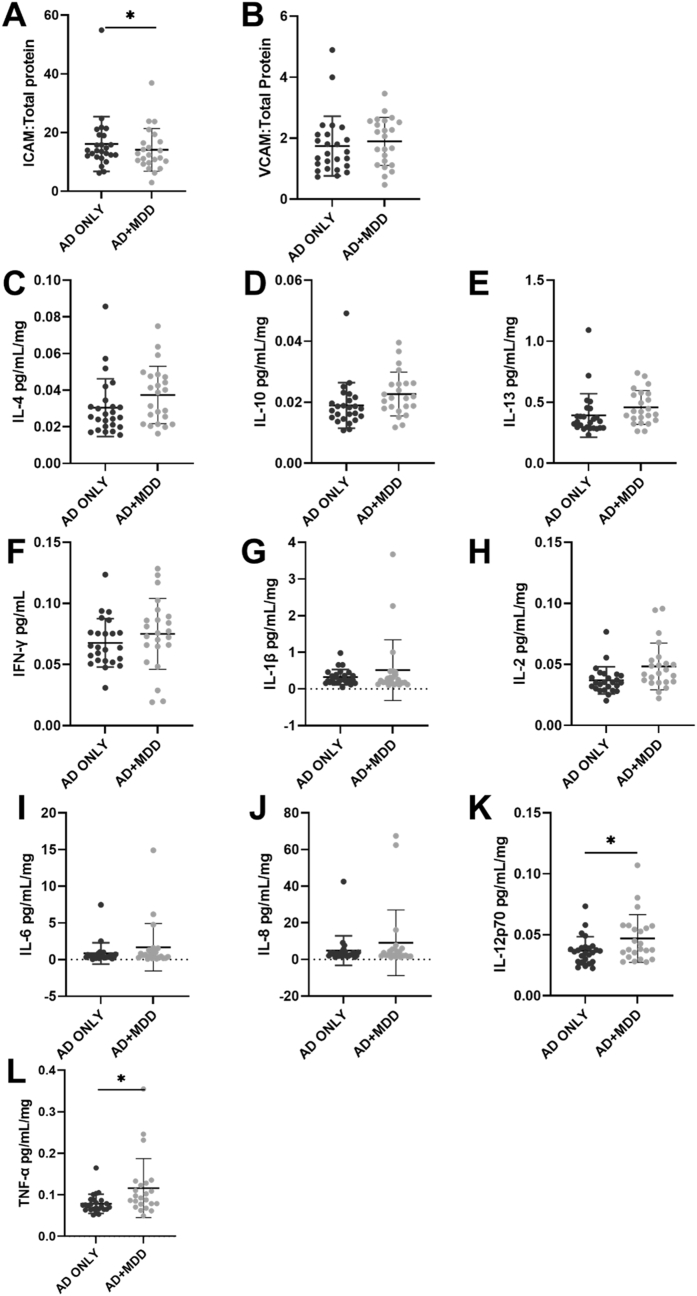
Expression of endothelial cell activation markers and cytokines in the insular cortex of AD and AD + MDD groups. ICAM-1 (A) but not VCAM-1 (B) appeared to be increased in the AD alone group. There was no statistical evidence of a between group difference in anti-inflammatory cytokines in the insula (C–E). TNFα (L) and IL-12p70 (K) appeared to be increased in those with co-morbid depression and AD (L) but there was no between group difference in the other pro-inflammatory cytokines measured (F–J). Please note that all analyses included covariates except for IL-1β which was not normally distributed.

In the insula, no difference was detected for the anti-inflammatory cytokines (IL-4: F_1,37_ = 1.656, p = 0.206, IL-10: F_1,37_ = 2.409, p = 0.129 and IL-13: F_1, 37_ = 0.551, p = 0.462, Fig. 2C–E), indicating that depression does not impact the anti-inflammatory cytokine expression in this area. Some of the pro-inflammatory cytokines were more highly expressed including: IL-12p70 (F_1,37_ = 4.185, p = 0.048*, Fig. 2K) and TNFα (F_1,37_ = 5.096, p = 0.03*, Fig. 2L) in AD + MDD group (IL-12p70: 0.047 ± 0.02 and TNFα: 0.116 ± 0.071) compared to the AD only group (IL-12p70: 0.037 ± 0.02 and TNFα: 0.078 ± 0.023), suggesting that co-morbid depression in AD is associated with increased pro-inflammatory cytokines release in this brain area.

### 3.2 Endothelial cell activation

In the superior frontal gyrus medial segment, none of the endothelial cell activation markers were different between AD + MDD and AD only groups (ICAM-1: F_1,34_ = 0.072, p = 0.791 and VCAM-1: F_1,34_ = 0.422, p = 0.52, Fig. 1A–B), indicating that MDD did not have an additive effect in terms of endothelial inflammation.

In the insula, a higher level of ICAM-1 was detected in the AD only group (16.109 ± 9.339, F_1, 37_ = 5.186, p = 0.029, Fig. 2A) compared to AD + MDD group (14.106 ± 7.285), which was not observed for VCAM-1 expression (F_1, 37_ = 0.082, p = 0.776).

### 3.3 Microglial markers

**Fig. 3:**
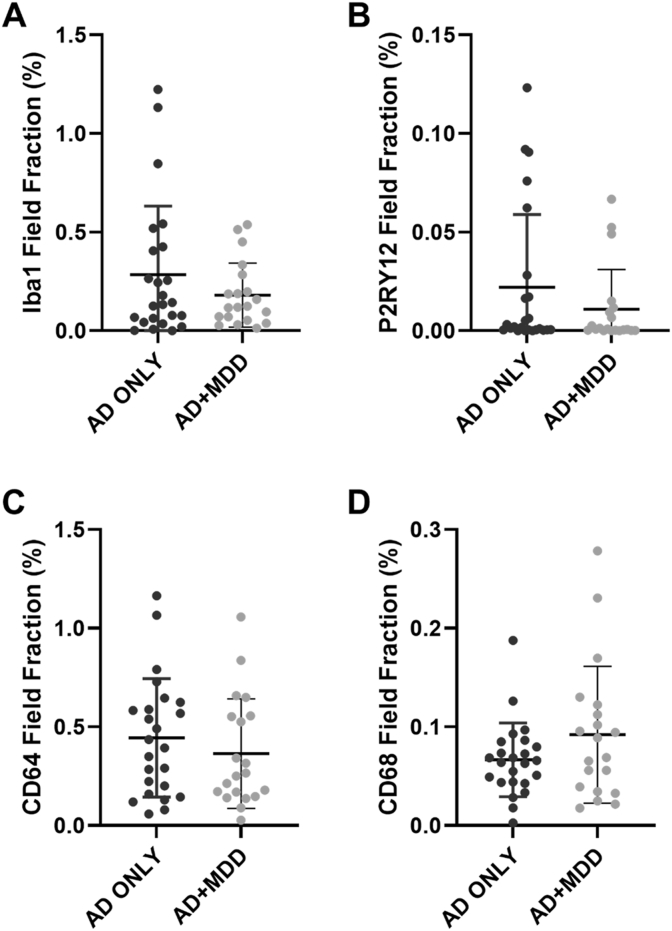
Quantification of the markers of microglial function in post-mortem brain tissue (superior frontal gyrus) of those with depression and AD compared to AD alone. Box plots showing the percentage field fraction of the four markers of microglial function investigated with immunohistochemistry. AD = Alzheimer's disease. There was no evidence of a between group difference in any of the markers of microglia (A–D).

**Fig. 4:**
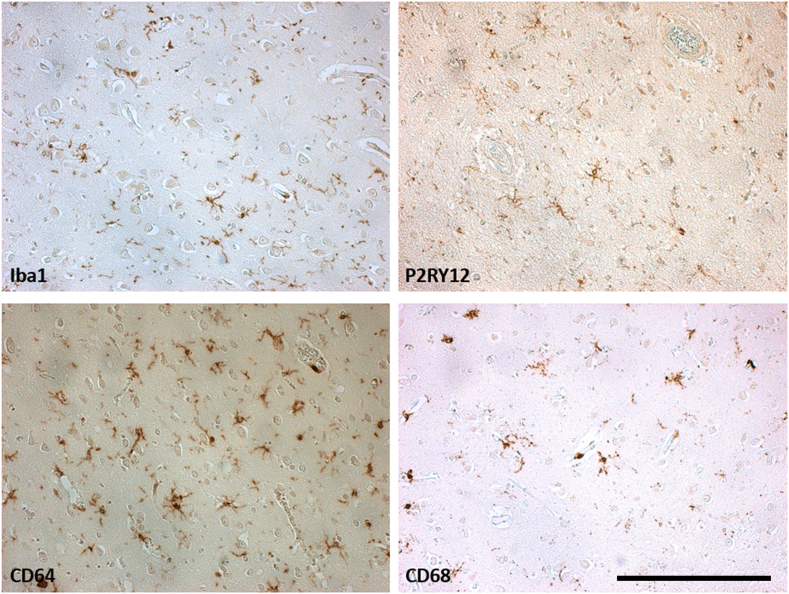
Representative examples of the microscope view of the immunohistochemistry staining obtained for the different microglial markers in individuals with AD included in this study. Counterstaining: Haematoxylin. Magnification ×200, scale bar = 250 μm.

Quantification of the microglial markers performed in the superior frontal gyrus medial segment showed no differences between the AD + MDD compared to the AD only groups (Fig. 3 & Fig. 4). Immunohistochemistry was not performed in the insula due to these overwhelmingly null findings and because of budgetary constraints.

## 4 Discussion

In this *post-mortem* brain tissue pilot study investigating the link between depression and inflammation in AD, we found changes in both pro and anti-inflammatory markers. This may reflect homeostatic control mechanisms such as the anti-inflammatory cytokine IL-10 increasing in acute inflammation as well as TNFα. Specifically IL-4 was increased in the AD + depression group in the superior frontal gyrus and that IL-12p70 and TNFα were increased in the anterior insula. A decrease in ICAM-1 expression in the anterior insula was seen in the AD + depression group. We found no between group differences in the markers of microglial function in the superior frontal gyrus.

Both depression and AD are associated with the presence of neuroinflammation, although the majority of evidence comes from studies of peripheral markers and the direction of causation remains unclear. In a preclinical model, treatment by Ibrutinib, a tyrosine kinase inhibitor which reduces LPS-induced neuroinflammation, attenuates synaptogenesis dysfunction and improves depressive like behaviour in a depression mouse model (Li et al., 2021), suggesting that neuroinflammation plays a role in the pathophysiology of depression (Li et al., 2021; Al-Ghraiybah et al., 2022; Ting et al., 2020). It has been acknowledged that depression and AD are associated with increased neuroinflammation, but the immune mechanism may be different. Immune response activation may not be chronic, baseline cytokines may not be significantly different among groups, but they may be affected to different degrees.

We found IL-4 to be increased in the AD with depression group in the superior frontal gyrus. IL-4 is a multifunctional cytokine found in the brain and associated with an immunosuppressive environment (Rakic et al., 2018). It regulates inflammation and has neuroprotective implications through inducing anti-inflammatory phenotype microglia (Zhang et al., 2021). Studies in AD and depression have shown discrepancies in IL-4 expression (Liu et al., 2020; Strawbridge et al., 2017; Osimo et al., 2020; Kang et al., 2015). This highlights the heterogeneity of inflammatory markers found in depression and AD in the current literature and the need for further high-powered studies to clarify any differences. Patients with mild cognitive impairment (MCI, n = 20) were found to have higher peripheral IL-4 compared to controls (n = 20), however increased dementia disease severity was associated with decreased plasma IL-4 potentially representing a more chronic inflammatory environment (King et al., 2018). This decrease in IL-4 with dementia severity may help explain the rapid cognitive decline seen in later stages of dementia as IL-4 can protect against amyloid-beta (Aβ)-induced neuronal toxicity while inducing a microglial phenotype that favours brain homeostasis and neuronal protection and repair (Szczepanik et al., 2001; Cherry et al., 2015). The IL-4 effects on microglia also show benefit in depression where infusion of IL-4 alleviated IL-1β induced depressive behaviours in rats (Jia et al., 2021). A recent study in mice reveals that suppression of IL-4 can increase vulnerability to stress and prevalence of depressive-like behaviours (Zhang et al., 2021). Furthermore, up-regulation of IL-4 triggered BDNF-dependent neurogenesis and protected against stress vulnerability and depressive behaviours (Zhang et al., 2021).

One *post-mortem* study comparing cytokine changes in the superior frontal gyrus reported significant increases in IL-4 and IL-5 levels, whereas no changes were observed in IL-6 and IL-10 levels in the prefrontal cortex of AD patients (Tennakoon et al., 2022). In our study we also found differences between the two brain areas under investigation, which may be related to the different roles that they play in cognitive and emotional function. For example, it has been suggested that the insula acts to integrate the functions of other brain areas and may form part of the default mode network (Sliz and Hayley, 2012). It is also possible that the differences seen are part of the homeostatic control mechanism, as discussed previously.

Patients with moderate to severe AD have been previously shown to have elevated serum IL-2 levels which correlated with dementia severity (Huberman et al., 1995). Another study found that increased IL-2 levels induced the release of TNF-α and IFN-γ from natural killer cells within the serum of AD patients, representing a chronic pro-inflammatory state (Solerte et al., 2000). Higher plasma concentrations of IL-2 and IL-6 in depression have been reported, although in our study we did not find any between group differences in IL-2 (Maes et al., 1995; Liu et al., 2012). Increases in serum and CSF TNF-α levels have been one of the most consistent findings in AD and depression pathologies (Solerte et al., 2000; Swardfager et al., 2010). In our study we found an increase in TNF-α in the anterior insula in AD with depression, but not in the superior frontal gyrus. One study reported a significant increase in the serum levels of TNF-α, IL-1β and IL-6 in patients with AD and depression, compared to AD patients without depression, with strong inverse correlations between the MMSE scores and pro-inflammatory cytokine levels (Khemka et al., 2014). A recent meta-analysis reported that TNF-α and IL-6 levels were increased in CSF in MDD (Enache et al., 2019). Higher levels of TNF-α have been associated with accelerated cognitive decline and mediate induction of depressive-like symptoms in humans and animal models (Marioni et al., 2010; Hennessy et al., 2017; Cheng et al., 2015).

Our study is the first to explore the microglial immunophenotype in the context of co-morbid depression in AD. Microglia are highly changeable cells regarding their function, varying in response to cytokine expression (Zhang et al., 2021). Proinflammatory signals induce activated response microglia to produce proinflammatory cytokines including TNF-α, IL-1β and IL-6 (Jia et al., 2021). Alternatively, activation of microglia can be induced by IL-4 and IL-13 and they then produce further anti-inflammatory cytokines, including IL-4, IL-10 and IL-13 (Jia et al., 2021).

We found no evidence for alterations in microglial expression in the superior frontal gyrus between the two groups. This observation implies that in the late stage of the disease, the presence of depression does not appear to affect the microglial function in AD associated with motility (Iba1, P2RY12) or phagocytosis (CD68). Of note, although we did not observe difference in the homeostatic microglial marker P2RY12, a previous study reported an increase of this marker in depression in those without dementia (Böttcher et al., 2020) and a mouse model of postpartum depression suggested that decreased hippocampal expression of P2RY12 is associated with depressive symptoms (Kim et al., 2023). In AD without depression and compared to controls, Iba1 and P2RY12 expression were reported unchanged (Asby et al., 2021; Franco-Bocanegra et al., 2019), while CD68 and CD64 expression was increased (Asby et al., 2021). Interestingly, these microglial markers were not modified by the presence of systemic infection (Asby et al., 2021), as observed in our study. Microglia in AD express increased CD64 and are known to lose their motility, represented by a decrease in Iba1 (Minett et al., 2016); however, no existing evidence for a significant change has been shown for these markers in depression. We would like to highlight that while our cohorts of cases remain one of the largest for the study of depression in AD, there is a large heterogeneity in the microglial response within each cohort that might impact on the power of the study and thus additional cases would be welcome. Also it is worth mentioning that in this pilot study, only four microglial markers were used and that in the systemic infection study in AD, the microglial changes were observed with the anti-inflammatory markers (Asby et al., 2021). Therefore additional markers are needed to carry on the immunocharacterisation of the microglial phenotype in AD with co-morbid depression.

The role of vascular endothelial cells as both participants and regulators of inflammation is increasingly recognised (Pober and Sessa, 2007). We investigated the expression of the cerebrovascular endothelial inflammatory markers ICAM-1 and VCAM-1. These cell adhesion molecules are suppressed in resting endothelial cells but increased by induction via pro-inflammatory cytokines (Pober and Sessa, 2007). We found no between group differences in either ICAM-1 or VCAM-1 in the superior frontal gyrus and no between group differences in VCAM-1 in the insula. We did however find reduced ICAM-1 in the insula in those with co-morbid depression. Our results are consistent with another *post-mortem* study examining white-matter changes in late-life depression showing increased ICAM-1 concentration with no significant differences in VCAM-1 concentration in the dorsolateral prefrontal cortex (Thomas et al., 2001). Our results imply that depression in AD is unlikely to cause a change in endothelial inflammatory function. In contrast one plasma-based study reported that elevated serum levels of ICAM-1 had a strong positive correlation with hopelessness and a negative correlation with cortical thickness in the inferior temporal gyrus in depressed subjects (Tsopelas et al., 2011; Chi et al., 2014).

Both markers of cerebrovascular function have been shown to be increased in CSF in AD (Asby et al., 2021; Janelidze et al., 2018). CSF ICAM-1 levels showed a positive correlation with increased risk of developing AD (Janelidze et al., 2018) and *post-mortem* tissue studies have shown increased ICAM-1 expression in areas with AD pathology (Akiyama et al., 1993). Previous studies looking into depression, not in the context of AD, have reported increased ICAM-1 (Müller, 2019). However, the significance of a difference in ICAM-1 may differ depending on the brain area with one study showing increased ICAM-1 expression in the dorsolateral prefrontal cortex which was not observed in the anterior cingulate cortex and occipital cortex (Thomas et al., 2004), consistent with our observations of a different expression between the superior frontal gyrus and the insula. The same study found no difference in VCAM-1 between depression and controls for multiple brain areas (Thomas et al., 2004).

Numerous studies have investigated the inflammatory environment in depression and AD separately. The uniqueness of the present study lies in the investigation of inflammation in both conditions and the measurement of neuroinflammatory markers in human brain tissue. We have investigated a variety of inflammatory components including microglial markers, endothelial inflammation markers and cytokines, giving a more holistic view of the inflammatory response. Further strengths of this study include the use of *post-mortem* tissue, as AD can currently only be confirmed pathologically *post-mortem*, allowing us to study the disease in its complexity.

However, the use of *post-mortem* tissue limits us to a retrospective, observational study. Therefore, we only assess the consequences of AD and depression at the time of death and could not study changes over time, limiting our ability to interpret causality or underlying mechanisms. As this was a pilot study it was not possible to perform a power calculation prior to the study. A post-hoc power calculation, using IL-13 as an example of reaching significance, revealed that we only had 43% power to detect a 1.2X mean difference and, to raise this to a power of 80% at the 95% confidence interval, would have required a sample size of 108 cases, more than double the available sample size for this study (Dean et al.). No correction was performed for multiple testing and thus it is possible that some of the differences observed between groups may be due to a type I error. Individuals in the depression in AD were less likely to have suffered from a co-morbid inflammatory illness than the AD no depression group. The presence/absence of a co-morbid inflammatory illness was included as a co-variate in the analyses for the normally distributed data, but this was not possible where the data was not normally distributed. Finally, although we chose to focus on individuals with AD with and without depression, as both diseases have been studied singly and compared to controls, the lack of a control group without either AD or depression is a limitation of this study.

### 4.1 Conclusions

In this study, we report evidence of changes in both pro and anti-inflammatory cytokines in the brain in co-morbid depression in AD. We found no between group differences in microglial function in the superior frontal gyrus and no evidence of increases in endothelial inflammation in those with depression in AD. This may represent regional differences in neuroinflammation in co-morbid depression in AD. Future studies should aim to obtain tissue from greater numbers of individuals and to examine a wider range of brain areas.

## Ethical approval

This work was carried out under the South West Dementia Brain bank generic ethical approval (ref 18/SW/0029).

## Data availability

All clinical data used in this project is available to bona fide researchers who register with the UK Brain Bank database (https://brainbanknetwork.ac.uk/). Data from the biochemical analyses are available upon reasonable request from the corresponding author, subject to the relevant permissions.

## Declaration of competing interest

The authors declare the following financial interests/personal relationships which may be considered as potential competing interests:Dr Lindsey Sinclair, Dr Mizuki Morisaki reports financial support was provided by Alzheimer's Society. Professor Golam Khandaker reports financial support was provided by Wellcome Trust. Professor Golam Khandaker reports was provided by UKRI Medical Research Council. Professor Golam Khandaker reports financial support was provided by National Institute of Health Research Bristol Biomedical Research Centre. Dr Lindsey Sinclair, Dr Mizuki Morisaki reports financial support was provided by University of Bristol Elizabeth Blackwell Institute for Health Research. None.
