# Supplementary material for: Neuroinflammation in comorbid depression in Alzheimer's disease: A pilot study using post-mortem brain tissue
Source: Neurosci Appl. 2024 Mar 3;3:104051. doi: 10.1016/j.nsa.2024.104051 (PMC11543635; doi:10.1016/j.nsa.2024.104051)
Supplement: Multimedia component 1 [file mmc1.docx]

Supplementary Information

**Table 1: Primary and Secondary antibodies for Immunohistochemistry**

| Antibody | Incubation Time | Species | Dilution | Provider |
| --- | --- | --- | --- | --- |
| Iba1 polyclonal | Overnight | Rabbit | 1:1500 | Wako, AlphaLabs (019-19741) |
| CD68 clone  PG-M1 | Overnight | Mouse | 1:50 | Dako (M0876) |
| CD64 polyclonal | 90 minutes | Goat | 1:100 | R&D systems (AF1257) |
| P2RY12 polyclonal | 90 minutes | Rabbit | 1:500 | Sigma Aldrich (HPA014518) |
| Biotinylated Universal Secondary Antibody (anti-rabbit/mouse IgG) | 20 minutes | Horse | Already made up | Vector Laboratories  (PK-7800) |
| Biotinylated, universal pan-specific antibody (anti-mouse/ rabbit/ goat IgG) | 20 minutes | Horse | 4drops horse serum  2drops of secondary antibody  2ml PBS | Vector Laboratories  (PK-8800) |

**Supplementary Table 2: Demographic and clinical characteristics of the depression and AD group**

| Case No. | Sex | Age of Death | Cause of death | Duration of AD | Braak stage | APOE genotype | PMD (hours) |
| --- | --- | --- | --- | --- | --- | --- | --- |
| 1 | M | 81 | Bronchopneumonia | 15 | VI | 4.4 | 4 |
| 2 | F | 80 | Bronchopneumonia, frailty, dementia | 9 | V | 4.4 | 50.75 |
| 3 | F | 88 | Bronchopneumonia, dementia | 11 | VI | 3.3 | 64 |
| 4 | M | 65 | AD | 10 | V | 3.3 | 38.5 |
| 5 | M | 64 | AD | 10 | V | 4.4 | 66.5 |
| 6 | F | 84 | AD | 4 | V | - | 22 |
| 7 | F | 86 | Urinary tract sepsis | 12 | VI | - | 45.25 |
| 8 | M | 92 | Dementia | 13 | V | 3.3 | 30.5 |
| 9 | M | 71 | Bronchopneumonia, AD | 10 | VI | - | 62.5 |
| 10 | M | 84 | AD | 8 | V | - | 13.75 |
| 11 | M | 94 | Frailty of age, AD | 3 | V | 3.4 | 45.5 |
| 12 | M | 67 | Bronchopneumonia, AD | 11 | VI | - | 36 |
| 13 | F | 95 | Frailty, Dementia | 5 | III | 2.4 | 65.5 |
| 14 | M | 70 | Cerebral infarct, dementia | 12 | V | 3.3 | 32.25 |
| 15 | M | 63 | Corticobasal degeneration | 7 | VI | 3.4 | 21.25 |
| 16 | M | 80 | Dementia, liver disease | 7 | IV | - | 68.5 |
| 17 | F | 77 | Bronchopneumonia, AD | 3 | VI | 3.4 | 68.5 |
| 18 | M | 80 | Myocardial infarction | 7 | V | 3.4 | 43 |
| 19 | F | 89 | Logopenic progressive aphasia | 11 | V | 3.3 | 35.75 |
| 20 | F | 81 | AD | 6 | V | - | 42 |
| 21 | F | 82 | Not stated | 10 | V | 2.4 |  |
| 22 | F | 67 | Bronchopneumonia, AD | 6 | VI | 4.4 | 24.25 |
| 23 | M | 69 | AD | 11 | VI | 3.4 | 7.75 |

APOE genotyping was not available for all cases. PMD post-mortem delay, AD Alzheimer’s disease.

**Supplementary Table 3: Demographic and clinical characteristics of the AD alone group**

| Case No. | Sex | Age of Death | Cause of death | Duration of AD | Braak stage | APOE genotype | PMD (hours) |
| --- | --- | --- | --- | --- | --- | --- | --- |
| 1 | M | 88 | Bronchopneumonia, AD | 3 | VI | - | 28 |
| 2 | F | 84 | AD | 6 | V | 2.4 | 19.5 |
| 3 | M | 86 | Old age, AD | 4 | VI | 2.3 | 19.5 |
| 4 | F | 88 | Frailty, dementia | 7 | VI | - | 7.5 |
| 5 | M | 77 | Bronchopneumonia, AD | 13 | VI | - | 21.75 |
| 6 | M | 81 | Dementia | 8 | VI | - | 32 |
| 7 | M | 95 | Dementia | 3 | III | 3.3 | 25.45 |
| 8 | F | 99 | Old age, dementia | 8 | V | 2.3 | 43 |
| 9 | F | 83 | Metastatic colorectal cancer |  | V | 3.4 | 30.5 |
| 10 | M | 81 | Dementia | 9 | V | 3.4 | 55.5 |
| 11 | F | 87 | Dementia | 3 | IV | 3.3 | 15 |
| 12 | F | 81 | Pneumonia, dementia | 15 | VI | - | 6.25 |
| 13 | M | 70 | AD | 13 | VI | 3.3 | 26.75 |
| 14 | M | 82 | Cardiac failure | 1 | III | 3.4 | 51.25 |
| 15 | F | 66 | Dementia | 5 | VI | 3.3 | 37.25 |
| 16 | F | 87 | Pneumonia, AD |  | V | 3.3 | 40.5 |
| 17 | M | 85 | Aspiration pneumonia, dementia | 11 | VI | 3.3 | 31 |
| 18 | F | 84 | Subarachnoid haemorrhage | 1 | VI | 3.3 | 10.25 |
| 19 | F | 73 | Corticobasal degeneration | 4 | VI | 3.4 | 25.75 |
| 20 | M | 80 | Frailty, AD | 12 | VI | 2.4 | 21.25 |
| 21 | F | 86 | Chronic lymphocytic leukaemia, AD | 5 | V | 2.4 | 37.5 |
| 22 | F | 85 | AD | 11 | VI | 3.4 | 17.75 |
| 23 | F | 93 | Frailty of age | 11 | V | - | 46.75 |
| 24 | F | 68 | Chest infection | 12 | VI | - | 46.25 |
| 25 | F | 81 | AD | 4 | V | - | 58.42 |

*APOE* genotyping was not available for all cases. PMD post-mortem delay, AD Alzheimer’s disease.
